# Physical characteristics not psychological state or trait characteristics predict motion during resting state fMRI

**DOI:** 10.1038/s41598-018-36699-0

**Published:** 2019-01-23

**Authors:** Hamed Ekhtiari, Rayus Kuplicki, Hung-wen Yeh, Martin P. Paulus

**Affiliations:** Laureate Institute for Brain Research (LIBR), Tulsa, OK USA

## Abstract

Head motion (HM) during fMRI acquisition can significantly affect measures of brain activity or connectivity even after correction with preprocessing methods. Moreover, any systematic relationship between HM and variables of interest can introduce systematic bias. There is a large and growing interest in identifying neural biomarkers for psychiatric disorders using resting state fMRI (rsfMRI). However, the relationship between HM and different psychiatric symptoms domains is not well understood. The aim of this investigation was to determine whether psychiatric symptoms and other characteristics of the individual predict HM during rsfMRI. A sample of n = 464 participants (174 male) from the Tulsa1000, a naturalistic longitudinal study recruiting subjects with different levels of severity in mood/anxiety/substance use disorders based on the dimensional NIMH Research Domain Criteria framework was used for this study. Based on a machine learning (ML) pipeline with nested cross-validation to avoid overfitting, the stacked model with 15 anthropometric (like body mass index, BMI) and demographic (age and sex) variables identifies BMI and weight as the most important variables and explained 10.9 percent of the HM variance (95% CI: 9.9–11.8). In comparison ML models with 105 self-report measures for state and trait psychological characteristics identified nicotine and alcohol use variables as well as impulsivity inhibitory control variables but explain only 5 percent of HM variance (95% CI: 3.5–6.4). A combined ML model using all 120 variables did not perform significantly better than the model using only 15 physical variables (combined model 95% confidence interval: 10.2–12.4). Taken together, after considering physical variables, state or trait psychological characteristics do not provide additional power to predict motion during rsfMRI.

## Introduction

Head motion (HM) during scanning can have profound effects on resting-state functional magnetic resonance imaging (rsfMRI) data^1^. A small, but significant, amount of this noise can remain in the data after conventional preprocessing pipelines^2–4^. Recent investigations have shown that motion related artifact can introduce a systematic bias in rsfMRI connectivity measures^5–7^. Therefore, systematic bias can emerge in studies where there are relationships between motion inside the scanner and variables of interest, like group (healthy control vs. a clinical population) or a disease severity^8^. There is a growing concern about the differential effect of HM on connectivity or activation studies in different clinical populations such as autism^9,10^, different subgroups of autism^11^, attention deficit/hyper activity disorder (ADHD)^10,12^, bipolar disorder and schizophrenia^13^. Moreover, this concern extends to structural neuroimaging studies, which have also found evidence for population-specific systematic bias in HM for T1-imaging^10,14^ and diffusion tensor imaging^15^. In comparison, much less is known about which physical and psychological characteristics have the most profound effects on HM.

Several test-retest rsfMRI studies showed a moderate level of intra-individual stability in the amount of HM and raised the potential contribution of both state and trait variables^7,16,17^. Four recent genetic studies also reported a moderate level of heritability for motion in various samples with different age (mean age range from 21 to 43 in adults and 10–11 in children), ethnicity and level of shared genetic backgrounds^17–20^. Several recent investigations have examined some of the specific trait or state factors influencing motion. Various studies show that men have more head motion than women^10,13^ and there is more motion with increasing age in adults^10,13^ and less motion with increasing age in children^10^. BMI is also consistently reported as a trait, or a relatively stable state, correlated with motion^18,21,22^. Both self-report^12^ and behavioral measures of impulsivity^18^ are reported as correlates of motion. In a seminal work in a subsample of 457 healthy adults from the Human Connectome Project, Siegel, *et al*.^22^, reported 23 out of 122 behavioral, demographic and physiological variables including BMI, Tobacco use and externalizing symptoms to be correlated with motion^22^. However, the influence of several other exploratory domains on motion during MRI scanning has been less well explored. Specifically, other factors that can have potential contribution to motion inside the scanner include positive and negative affective states and traits, physical activity, interoceptive awareness, sleep disorders, fatigue, pain, hunger, emotion dysregulation, personality traits like extraversion or neuroticism, history of psychological trauma, different aspects of self-control, and legal or illegal drug use profile including severity of dependency or withdrawal. Moreover, anthropomorphic variables other than BMI and weight such as height, hip size and body composition, i.e., percent of fat, muscle or water, have not been explored. However, the high number of potential predictors for motion and shared variance among some of them add to the complexity of this exploration and necessitate recruitment of more sophisticated multivariate statistical models. Some of the previous findings in the field were concluded from massive univariate analyses, which raised the concern about reproducibility^23^.

This investigation aimed to use a machine learning (ML) framework to (1) identify which physical or psychological variables best predict HM during rsfMRI and (2) to determine whether physical characteristics or psychological state/trait variables are better predictors of HM. We used n = 464 subjects from the Tulsa1000 (T1000) cohort with a wide range of dimensional phenotypes for mood, anxiety and substance use from healthy to different levels of pathologic severity^24^ to evaluate the degree to which the experimenter can use prior information to predict motion during scanning on an individual subject level. Understanding the variables that predict HM can help to (1) provide prospective instructions to participants to minimize motion, (2) elucidate whether systematic difference in variables across target populations contribute to neuroimaging differences, and (3) help to identify possible covariates for subsequent analyses.

## Methods

### Participants

Data were collected from 464 participants (174 male) from the T1000, a naturalistic longitudinal study recruiting subjects based on the dimensional NIMH Research Domain Criteria framework for mood/anxiety and substance use disorders. In the T1000, we recruit subjects between 18 and 55 years of age (mean age = 35.3, standard deviation = 10.5, range 18–56 at time of scanning) (more details about the subjects are provided in supplement Table S1). Subjects are recruited from local treatment facilities or from people seeking treatment for anxiety and/or depressive symptoms or substance use disorders. Other than routine exclusion criteria for fMRI studies, a positive test for drugs of abuse, including alcohol (breath test), cocaine, marijuana, opiates, amphetamines, methamphetamines, phencyclidine, benzodiazepines, barbiturates, methadone, and oxycodone, or having any of the following DSM-5 disorders: schizophrenia spectrum and other psychotic disorders, bipolar and related disorders, obsessive-compulsive and related disorders, or any changes during last 6 weeks in the medications that affects brain functions including antidepressants, anxiolytics, antipsychotics or mood stabilizers were considered as exclusion criteria. Ethical approval was obtained from Western Institutional Review Board T1000 protocol #20142082. All methods were carried out in accordance with relevant guidelines and regulations. Informed consent was obtained by members of the research team that have received training from the PI (MPP) to obtain consent for this study.

### Data collection procedure

Participants underwent an intensive assessment for demographic, anthropomorphic, clinical and psychiatric features, with a main focus on mood, anxiety, trauma, drug use, personality, social and physical function, disability, sleep, fatigue, pain, impulsivity and eating dimensions. From this assessment, we used 120 direct and derived variables. The list of these 120 variables is provided in supplemental Table S2 with details.

Anthropometric body composition variables were assessed using an InBody370 Impedance Body Composition Analyzer (InBody Co., Ltd., South Korea). This device uses 15 impedance measurements (3 frequencies: 5 kHz, 50 kHz, 250 kHz; five body segments: right arm, left arm, trunk, right leg, left leg) to produce highly accurate composition estimates.

Resting state functional magnetic resonance imaging (rsfMRI, parameters: TR = 2000ms, TE = 27 ms, FOV = 240 mm × 240 mm, 2.9 mm slice thickness, 128 × 128 matrix, 39 axial slices, and 240 repetitions) was performed using two identical GE Discovery MR750 3 T scanners while subjects fixated on a cross with eyes open and instructions not to think of anything in particular.

### fMRI data processing

Minimal processing was done in AFNI^25^. Specifically, head motion was estimated as six parameters using 3dVolreg (3 translations and 3 rotations) prior to any other processing, as there is some concern that slice-time correction may lead to underestimates of motion^26^. The Euclidean norm (ENORM) of the derivative of the six parameters was taken as a measure of motion per TR. This ENORM was averaged across the entire scan, producing a single metric of overall movement for each scan^25^. Finally, average ENORM motion was natural log transformed to deal with a right skew in the distribution. The log transform of the mean ENORM of the six motion parameter derivatives then served as the dependent variable in all prediction analyses.

ENORM is the main measure of motion in the AFNI analysis pipeline [25]. But, for audiences with more familiarity with Framewise Displacement (FD), we also measured FD. FD is defined as the sum of the absolute values of the six motion parameters. FD can also be called the L1 norm compared to ENORM that is the L2 norm (square root of the sum of the squared values). For comparison, we computed average FD (and natural log FD), and show in Supplement Figure S1 that in this dataset the correlation coefficient between average FD and average ENORM is greater than 0.99.

### Statistical modeling

Machine learning methods can identify features in the data useful in predicting outcome measures. However, different methods have different assumptions and may predict outcomes with different accuracy, and no single method can always make better predictions than other methods. One solution is to use the “wisdom of crowds” (Marbach *et al*., 2012) by combining predictions from multiple base learners (prediction algorithms). In this work, we (1) utilized multiple ‘out-of-box” machine learning methods, and (2) combined the predictions across methods by stacking or meta ensemble^27–29^. In order to assess performance of stack ensembles in independent, unseen datasets, we conducted nested cross-validation (nested CV) where the inner loop was used to build base and stacked models, and the outer loop to evaluate model performance (Supplemental Figure S2). We repeated the entire nested CV procedure 20 times to increase the stability of our results and estimate confidence intervals. Additionally, since some methods require complete data, any missing values in the 120 predictors were imputed using k-nearest neighbors before entering the repeated nested CV. This was done without considering motion, and no predictor was missing more than 6.25 percent (29 values).

#### Base learners

In the inner loop, we applied 6 base leaners for each training set: elastic net (ENET), principal component regression (PCR), partial least square (PLS), support vector regression (SVR), random forest (RF), and conditional inference forest (CF). For each method (base learner), the tuning parameter(s) were optimized by 10-fold cross validation (CV). Specifically, each training set was portioned into 10 distinct subsets, where 9 subsets were used for training process to make predictions on the remaining subset. Optimal hyper-parameter values were chosen by random search (Bergstra and Bengio, 2012) and the one-SE rule (Supplemental Figure S2b; Figure 3.7 of^30^) using ${R}^{2}$ as the model performance metric. Following this, we obtained 6 sets of predicted values, one from each base leaner, and their corresponding optimal hyper-parameter values.

#### Stacking ensemble

Within the inner cross validation loop, each method produced a single best model and expected out of sample ${R}^{2}$. A stacked model was built by taking the arithmetic mean of predictions from each base learner, weighted by each model’s expected out of sample ${R}^{2}$.

#### Assessment of the model performance

In the outer loop, the stacked model was applied to predict the response in the corresponding validation set. Predicted values of the validation sets were combined and compared with the observed values to compute ${R}^{2}$. With 20 replications of partitions, we summarized the performance by the mean and 95% confidence interval of ${R}^{2}$.

Additionally, we grouped predictor variables into categories of physical (demographics and anthropometrics) and psychological (non-drug related and drug related) variables and built different prediction models using different subsets of predictors to investigate their relationships with motion.

#### Variable importance

In addition to model performance, we also assessed variable importance (VI) using stacking. First, each base model provided importance for each feature. Note different individual methods had different VI measures: absolute values of regression coefficients for elastic net, weighted sum of absolute regression coefficients for PCR and PLS, “out-of-bag” mean square error obtained by permutation for RF and CF; for SVR, a “filter” approach (https://github.com/topepo/caret/blob/master/pkg/caret/R/filterVarImp.R) was used: the response variable was regressed on each feature one a time by a loess (LOcally WEighted Scatter-plot Smoother) and the R-square was computed as the variable importance. These VI measures were scaled to between 0 and 100 for each individual model. Next, the stacked importance was computed as the weighted average of the importance across models using the weights determined by the stacking model described above. This produced a single set of VI values for each stacked model in the outer loop of nested CV. VI was averaged across folds to obtain a single set of values. 20 random partitions were used (i.e. 20 repeats of nested CV), and 95% confidence intervals for VI were taken as each variable’s mean importance ±1.96 times its standard deviation.

We also selected a few predictors to demonstrate their relationship with motion by partial dependence plots^31^. In addition to the proposed machine learning approach, we also computed Pearson correlation coefficients, 95% confidence intervals and FDR corrected p-values for comparison purpose since massive univariate analyses are more common in the literature.

We implemented the analyses for prediction models using the caret package (version 6.0–76)^32^ and partial-dependence plots by the pdp package (Greenwell, 2017), and on R version 3.3.2.

## Results

**Figure 1:**
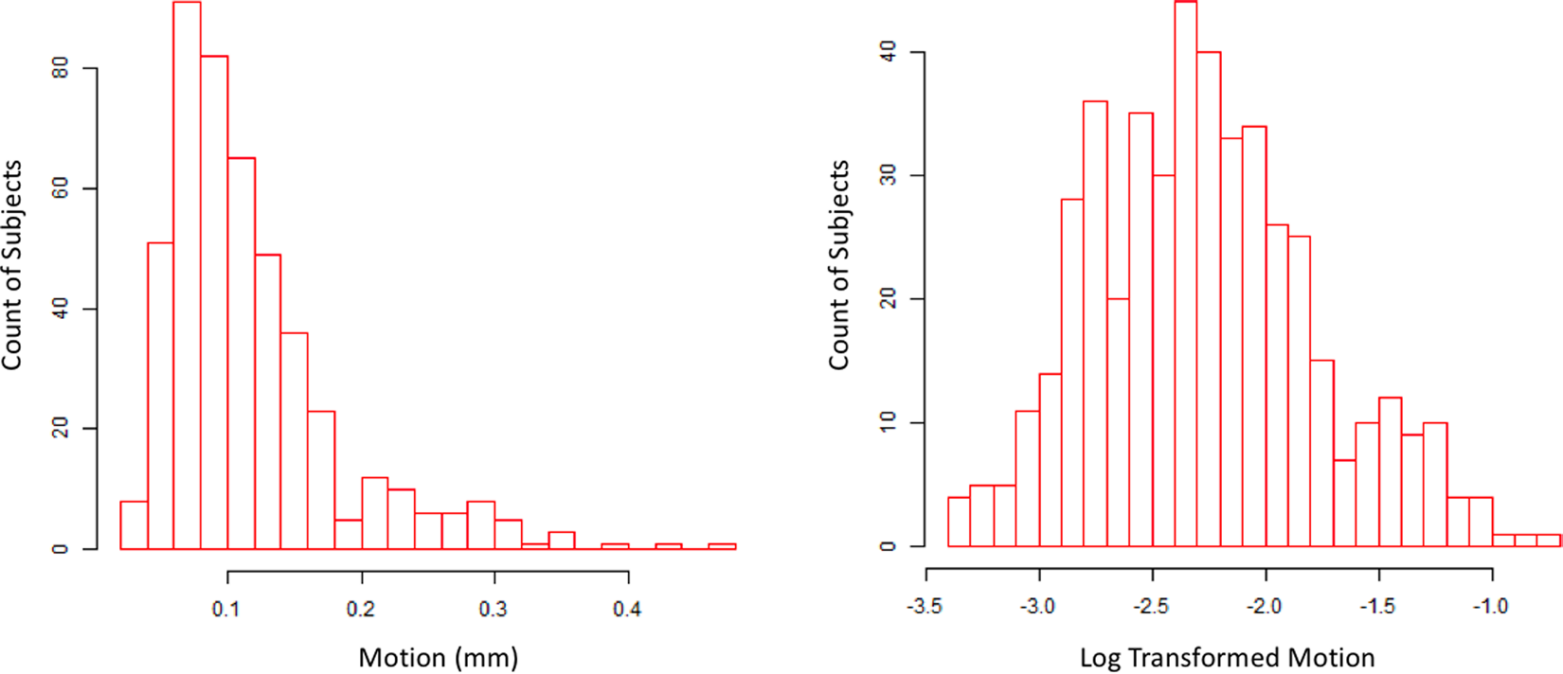
Distribution of motion (average Euclidean norm of six motion parameters) without and with log transformation (n = 464).

**Figure 2:**
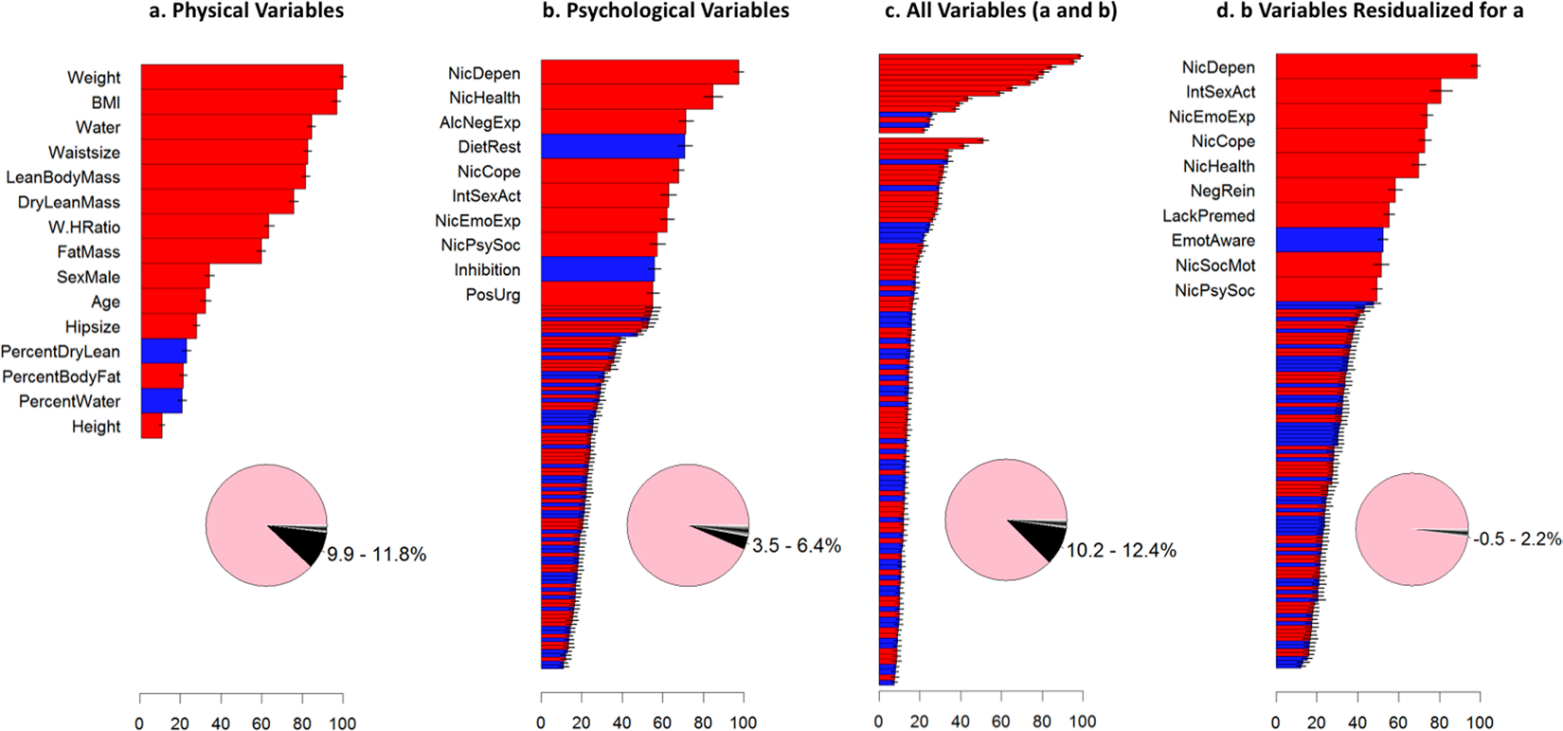
Variable importance (VI) for predicting motion and the corresponding R-squared. (**a**) Model with 15 physical (anthropometric and demographic) variables. Below pie chart depicts percent of variance explained by the model and its 95% confidence interval, (**b**) Model with 105 psychological (self-report) variables (first 10 variables with higher variable importance received broader bars for better visualization; find the complete list of variables and their VI in supplemental Table S2), (**c**) Adding psychological variables (from b, lower part of the graph) to physical variables (from a, upper part of the graph) did not substantially change the percent of variance explained by the model with only physical variables (**a**) and, (**d**) Model with psychological variables after removing linear effects of physical variables includes 0 in its 95% of confidence interval for percent of variance explained. VI is based on the stacked ensemble. Variables with bars in red or blue have a positive or negative univariate correlation with motion. Error bars represent 95% confidence intervals, taken across partitions. The standard deviation of these values was taken as an estimate of its standard error, and the mean +/− 1.96 times its standard error was taken as the 95% confidence interval. BMI: Body Mass Index, Water: Body Water Weight, W.HRatio: Waist Hip Ratio, NicDepen: PROMIS Nicotine Dependency score, NicHealth: PROMIS Nicotine Health Negative Expectancies score, AlcNegExp: PROMIS Alcohol Negative Expectancies score, DietRest: Three Factor Eating Questionnaire (TEFQ) Dietary Restraint Score, NicCope: PROMIS Nicotine Coping score, IntSexAct: PROMIS Interest in Sexual Activities score, NicEmoExp: PROMIS Nicotine Emotional and Sensory Expectancies score, NicPsySoc: PROMIS Nicotine Psychosocial Expectancies score, Inhibition: Behavioral Inhibition/Activation System (BIS/BAS) Inhibition score, PosUrg: UPPS Impulsive Behavior Scale (UPPS-P) Positive Urgency score, NegRein: Customary Drinking and Drug Use Record (CDDR) Negative Reinforcement score, LackPremed: UPPS Impulsive Behavior Scale (UPPS-P) Lack of Premediation score, EmotAware: Multidimensional Assessment of Interoceptive Awareness (MAIA) Emotional Awareness score NicSocMot: PROMIS Social Motivations for Nicotine score).

The distribution of HM of all participants is shown in Fig. 1 (left: original scale; right: log-scale). For all subsequent analyses, natural log transformed HM was used as the dependent measure. A model with physical (anthropometrics and demographic) variables explained 10.9% of variance (95% confidence interval 9.9–11.8%) (Fig. 2a, and supplemental Figure S5). Weight and BMI had the highest variable importance (VI) in this model, while height had the lowest VI. A model with all psychological variables explained 5% of variance (95% confidence interval 3.5–6.4%) (Fig. 2b). Among the top 10 variables with highest VI, 6 were related to drug use (5 nicotine-related and one alcohol-related), nicotine dependency score, nicotine health negative expectancies score, alcohol negative expectancies score, nicotine coping score, nicotine emotional and sensory expectancies score, nicotine psychosocial expectancies score (from PROMIS measures), all with positive correlation with motion (red bars in Fig. 2b). the other 4 variables are “impulse and control” related, two inhibition related variable s with negative correlation (blue bars in Fig. 2b): dietary restraint score from the three factors eating questionnaire (TEFQ)^33^, and inhibition score from behavioral inhibition/activation system (BIS/BAS) self-report^34^, and two impulsivity related variables with positive correlation with motion: positive urgency from the impulsive behavior scale (UPPS-P)^35^ and interest in sexual activities score from PROMIS (Patient-Reported Outcomes Measurement Information System)^36^.

Adding psychological variables to the model with physical variables did not significantly change the prediction accuracy (95% confidence interval 10.2–12.4%) (Fig. 2c, and supplemental Figure S5). This finding implies shared variance in HM between physical and psychological variables. To separate psychological from physical effects, we regressed each psychological variable on the 15 physical variables and used the residuals (“residualized psychological variables” hereinafter) as predictors in our ML pipeline. These residualized psychological variables explained less than 1% of motion variance and the corresponding 95% confidence interval contained zero (−0.5–2.2%) (Fig. 2d and supplement Figure S5), suggesting that variance explained by psychological variables is primarily due to shared variance with physical variables. In this model with residualized psychological variables, the majority of 7 variables out of top 10 variables with highest VI, were drug related (6 nicotine-related). Those 3 non-drug related variables, one is still interest in sexual activities score, and two others are still within the “impulse-control” domain: emotional awareness score from the multidimensional assessment of interoceptive awareness (MAIA)^37^ (negative correlation) and lack of premeditation, UPPS-P (positive correlation).

Previous investigations^22,38,39^ suggested that respiration rate and/or amplitude during scanning may mediate the relationships of weight and cigarette smoking with HM. Thus, we performed a secondary analysis to explore the potential mediation effects of respiratory rate and amplitude on the relationships between HM and weight and nicotine dependence, the most important physiological and psychological predictors. We found respiratory rate was associated with motion, but respiratory rate and amplitude did not mediate the relationships between weight and motion and between nicotine dependence and motion (please see supplementary materials and results for details).

## Discussion

In our sample comprising both healthy volunteer and several dimensionally defined psychiatric populations, we found that physical features but not psychological variables best predicted HM during rsfMRI. Moreover, nicotine and alcohol use severity and impulsivity/inhibition measures were the most important psychological variables predicting HM. However, psychological variables did not add significantly to the physical variables-based prediction of HM. These results add to the small but growing literature alarming about the shared variance between HM and other variables of interest in resting state functional connectivity studies^40^.

The ML models identified weight and BMI as the most important predictors of HM for both the physical predictor model only and the physical and psychological predictor model. Others have reported similar results previously using correlation analyses^18,21,22^. In our sample (mean age = 35.3, age range between 18 and 56), the Spearman correlation coefficient between HM (before log transformation) and BMI was 0.27 (95% confidence interval estimate 0.18–0.36, Pearson r = 0.28). Two studies with the Human Connectome Project (HCP) database in healthy participants (mean age = 28, age range between 22 and 37) reported higher level of correlation coefficient (Pearson r = 0.66 and Spearman r = 0.62, n = 461 and 863 participants)^18,22^ and one study in an older group of healthy adults (mean age = 43, age range between 18 and 85) from the Genetics of Brain Structure and Function (GOBS) database reported a similar level of correlation (Spearman r = 0.28, n = 606 participants)^18^. Other than simple measures of weight and height, we also measured more advanced body composition metrics. However, percent of body fat, water, or dry lean mass did not have significant correlation with HM, which suggest that participants with larger BMI are more likely to move inside the scanner, regardless the composition of body from fat, muscle or water. The preponderance of physical variables predicting HM is consistent with the notion that lack of enough space inside the scanner, higher respiratory rate and greater effort for respiration in the supine position may be potential causes. Siegel *et al*.^22^ suggested that respiration contribute in higher motion among people with high BMI but they did not have access to the respiration data in the Human Connectome Project database to test this hypothesis. To fill in the gap, we explored the mediatory role of respiration but did not find that respiratory rate or amplitude mediated the relationships between weight and HM. The contribution of BMI and weight in HM, independent to their type of relationship with HM, should be considered very carefully in any neuroimaging study that has variables of interests correlated with weight and BMI.

The model with 105 psychological variables only explains just 5% of variance in HM. However, this portion of the explained variance would be reduced to less than 1% when we linearly regressed out the 15 physical variables. Thus, adding psychological variables to an ML model based on physical characteristics of the subject does not aid to better predict HM. Among psychological variables, drug (including nicotine and alcohol) use related variables dominated in both models with and without regressing out the physical variables. Smoking has been previously reported to have a relationship with HM in both HCP and GOBS databases^18,22^. Lack of inhibitory control to remain still inside the scanner, withdrawal state and respiratory comorbidities like chronic obstructive pulmonary diseases, that may change the respiratory rate and amplitude, can be among explanations. However, in our secondary analysis, we did not find respiratory rate or amplitude mediate the relationships between nicotine dependence and HM. The relationship between nicotine dependence and HM inside the scanner is small but can be still an issue of concern for neuroimaging studies in clinical populations where use of nicotine is common.

We observed a negative relationship between HM and two self-control measures, (1) dietary restraint (with items like “*I often stop eating when I am not really full as a conscious means of limiting the amount that I eat*” or “*I consciously hold back at meals in order not to gain weight*”) and (2) behavioral inhibition (with items like “*I feel worried when I think I have done poorly at something important*” and “*I worry about making mistakes*”). In addition, there was a positive relationship between HM and (1) positive urgency (includes questions like “*When I am very happy, I can’t seem to stop myself from doing things that can have bad consequences*” or “*Others are shocked or worried about the things I do when I am feeling very excited*”) and (2) interest in sexual activities (asking questions like “*In the past 30 days how often were you interested enough to start a sexual activity?*”) support the role of impulsivity and inhibitory control in the level of HM inside the scanner. In the same direction, Kong, *et al*.^12^, reported that higher HM among children with ADHD is mediated by a self-reported impulsivity score. Furthermore, there might be an impulsivity core in both nicotine dependence and weight association with HM as well. However, any interpretation on shared variance between HM, nicotine/drug use, weight and impulsivity-related measures needs further study.

Our results show that physical and psychological variables had significantly overlapping explained variance for predicting HM. But, what is the implication of these finding for neuroimaging studies targeting these variables of interest? It is now well-established that HM can cause a systematic bias in functional connectivity measures, reducing connectivity between distant areas in the brain, like within default mode network (DMN) nodes, i.e., ventromedial prefrontal cortex (VMPFC) and posterior cingulate cortex (PCC)^16^. Some initial studies, which had started to consider the role of HM in making systematic bias in connectivity measures, found that previously reported lower distant connectivity among young vs. adult populations^5,6^ and elderly vs. adult populations^7^ were at least partially affected by this HM artifacts. Different studies in clinical populations, like autism^9^, reported similar observations, showing that controlling for HM can remove some previously recognized group differences in connectivity measures. Different scholars introduced various additional methods to remove this HM artifact more efficiently^2,3,41^. These efforts have not always been successful and satisfactory^3^. Siegel, *et al*.^22^, after using a comprehensive set of HM noise removal techniques reported that “our findings suggest that complete removal of the confounding influence of head HM is difficult or impossible”^22^. Results of this study, along with other recent studies on the correlates of HM^18,22^, alarm for a complex shared variance between (1) physical features like BMI, (2) psychological constructs like impulsivity/control and their associates like nicotine/alcohol/drug use, (3) HM and (4) distant connectivity within large scale networks like DMN or Executive Control Network (ECN)^21^. People can draw different causal relationship between these 4 dimensions but any causal inferencing from the available cross-sectional data even with more complex statistical methods like mediation analysis or structural equation modeling seems unjustified^42^. An optimal solution to tease apart this complex shared variance between physical and psychological variables and HM and connectivity measures seems farfetched. However, there is a growing body of indirect evidence that hopefully will provide a better picture of the problem that might be quite complex. For example, a study showed adding more HM-related variables as nuisance regressors to the resting state fMRI preprocessing will remove higher levels of network data with structures highly correlated to networks reported in the functional connectivity literature including the DMN^43^. Furthermore, in an interesting study design, using subjects with two sets of rsfMRI data, the authors reported that correlation between HM and distant connectivity in areas within DMN was stable independent of intra-individual differences in the level of HM across time. Based on this result suggesting biological basis for HM, the authors concluded that “head motion-associated differences in brain connectivity cannot fully be attributed to head motion artifacts but rather also reflect individual variability in functional organization”^16^. These studies show that we still need to be very careful of the implications of these finding for neuroimaging studies targeting variables of interest correlated with HM.

Based on our results and other published studies in the field^18^, we highly recommend researchers to be careful in over interpretation of the rsfMRI connectivity results when they have variables of interests in physical characteristics, impulsivity and drug use. Methods of HM nuisance removal should be carefully selected considering that even test retest reproducibility of large scale networks^44^ or group comparisons in functional connectivity are highly dependent on preprocessing strategies for HM correction^41,45^. There are hopes that multi echo fMRI sequences instead of conventional single echo pulse sequences can provide better opportunities for HM removal^39,46,47^. There are also potentials for prospective HM artifact removal using optical monitoring of HM with a marker attached to head^48^. However, the future of these methods does not seem very clear now. Interestingly, in our large sample of subjects with a wide range of mood/anxiety/substance-use disorders, different state and trait mood/anxiety/trauma related measures did not show any major contribution to the level of HM inside the scanner. These variables seem “safer options” for exploring covariates of functional connectivity for the resting state fMRI. Finally, traditional recommendations for reducing HM inside the scanner with better instruction to subjects, careful padding, giving feedback^49^ and controlling for state related variables such as nicotine withdrawal or hunger especially in people with trait-based vulnerability, like those who have higher BMI or are more prone to the lack of control like people with drug use problems, will still be very helpful.

This study has several limitations. First, there are different proxy measures of HM inside the scanner that are all correlated to each other^5,8^. Like Hodgson *et al*.^18^, we have used just one measure of HM in our study, computed as the average Euclidean norm of 6 HM derivatives. Pardoe *et al*.^10^, reported using different measures of HM “yielded very similar results”. But, Siegel, *et al*.^22^, reported both similarities and differences in correlates for various measures of HM. Another limitation of this study is using self-report measures of psychological constructs in relationship with behavioral measure of HM. There is a large body of evidence on the low level of shared variance between self-report and behavioral measures even within the same construct like impulsivity^50^. Having behavioral measures of psychological constructs might result in higher levels of shared variance with HM. The final limitation is lack of external validity testing for the models we developed in this study with another database. We have used repeated nested cross-validation to reduce the risk of over-estimation of prediction accuracy, but an external validation of the model with another independent sample will demonstrate its replicability.

## Conclusion

In this study, we found that physical characteristics dominate psychological characteristics in explaining HM during rsfMRI. We also found that there is shared variance between psychological and physical characteristics in explaining HM during resting state fMRI. Our results also show that nicotine/drug use and impulsivity/control might play a role in higher HM inside the scanner. The causal role of this shared variance between psychological or physical variables in explaining both HM and distant functional connectivity in large scale networks should be explored more in the future. Considering neuroimaging measures or psychological variables that are not correlated with HM, such as mood/anxiety/trauma measures, for clinical populations that are vulnerable to HM can be associated with more consistent results with higher specificity. Higher risk for HM inside the scanner among vulnerable individuals, like people with higher BMI or more impulsivity and nicotine/drug use problems, might be modifiable with more instructions and comfortable positioning on the scanner bed. Meanwhile, systematic examination of contextual contributions such as drug withdrawal, which can be modifiable, should be conducted before fMRI studies to reduce HM.

## Electronic supplementary material

Supplementary Materials

## Data Availability

The datasets generated during and analyzed during the current study are available from the corresponding author on reasonable request.
