# Supplementary material for: Physical characteristics not psychological state or trait characteristics predict motion during resting state fMRI
Source: Sci Rep. 2019 Jan 23;9:419. doi: 10.1038/s41598-018-36699-0 (PMC6344520; doi:10.1038/s41598-018-36699-0)
Supplement: Supplementary file 1 — Supplementary Materials [file 41598_2018_36699_MOESM1_ESM.pdf]

Supplementary Information

Title: Physical characteristics not psychological state or trait characteristics predict motion during resting state fMRI.

Authors: Hamed Ekhtiari, Rayus Kuplicki, Hung-wen Yeh, Martin P. Paulus.
Affiliations: Laureate Institute for Brain Research (LIBR), Tulsa, OK.

Corresponding Author: Hamed Ekhtiari, MD, PhD, Laureate Institute for Brain Research,
6655 South Yale Ave. Tulsa, OK 74136. hekhtiari@laureateinstitute.org, Phone No.: +1 918.502.5100

<u>Supplementary Materials</u>

Overview

1. Supplementary methods
    a. Machine learning methods
    b. Mediation analysis

2. Supplementary tables
    Table S1. Characteristics of 464 subjects recruited for this study.
    Table S2. A list of 120 psychological (self-reports, non-drug related and drug related) and physical (age and body composition) variables with both univariate correlation (Pearson, r and P-value FDR corrected) with motion (log transformed) and variable importance (VI) in the stacked model.
    Table S3. Pearson correlation coefficients between variables used in mediation analysis.
    Table S4. Path coefficients of mediation analyses.

3. Supplementary figures
    Figure S1. Scatterplots between two measures of motion, ENORM and FD.
    Figure S2. (a) the process of repeated nested CV; (b) the inner-loop of repeated nested CV.
    Figure S3. Correlation matrix between 120 variables.
    Figure S4. Percent of variance explained for motion with all variables using different methods.
    Figure S5. Percent of variance explained for motion with different sets of variables using combined (stacked) method.
    Figure S6. Partial dependence plots for the variables with the highest importance among anthropometric (a, BMI) and self-report (b, NicDepen) variables.
    Figure S7. Path diagram of the final mediation model.

1. Supplementary methods

a. Machine learning methods

<u>Selection of algorithms</u>. In this work, we implemented 6 algorithms: elastic net (ENET), principal component regression (PCR), partial least square (PLS), support vector regression (SVR), random forest (RF), and conditional inference forest (CF). The first three methods assume linear relationships between each predictor/feature and the response variable; ENET performs feature selection while PCR and PLS do not. PCR and PLS both assign weights on all features via orthogonal components, but the weights are determined in an unsupervised manner in PCR but supervised in PLS, thus the former tends to have larger bias but lower in prediction than the latter (James et al., 2013). The other three methods relax the linearity assumption between features and response variable by different means (various kernel functions in SVR, and recursive binary splits in RF and CF). Although CF (Hothorn, Hornik, & Zeileis, 2006) corrects the bias of RF in favor of variables with more distinct values (Strobl, Boulesteix, Zeileis, & Hothorn,

2007) in terms of variable importance, it's not clear how different the two methods are in prediction accuracy. For this reason, we included both forest methods.

Repeated nested cross-validation. It is well known that using the same dataset to build a prediction model and evaluate its performance can cause overestimation in the model performance (Varma and Simon, 2006). If we have two datasets, one of them can be used to train a prediction model, and the other to evaluate the model performance. Because we had only one dataset, one possibility was to split the data into a training set for model building and a validation set for model evaluation. Alternatively, the whole dataset could be partitioned to K_1 parts, where one part served as a validation set and the other parts were combined to serve as a training set, and the process iterated so that each part served as a validation set exactly once (Outer loop). For a given machine learning method, a training set could be partitioned into K_2 parts and the optimal tuning parameters could be determined by K_2-fold cross validation (Inner loop). The trained model was then applied to make predictions in the corresponding validation set. Iterating across the K_1 parts, the K_1 sets of predicted values were combined to compare with the observed values to evaluate model performance. The whole procedure is known as nested or double cross-validation (Stone, 1974; Varma and Simon, 2006), and was repeated by different partition indices to improve the stability of the results (Filzmoser et al., 2009). We chose to repeat 20 times of nested cross-validation (CV), with 10-fold CV for both the outer and the inner loops.

Stacked regression/Super learner (the inner loop)
In the inner loop, we applied 6 machine learning methods and then combined their predictions by stack ensemble. Suppose a training set $\mathbf{D}$ was expressed as a matrix, concatenated in columns by a response vector $\boldsymbol{y}$ and a predictor matrix $\mathbf{X}$, with $n \times (K_1 - 1)/K_1$ observations.
Step ❶ Building base learners. Each base learner was built by K_2-fold CV, which consisted of data partition and parameter optimization.

Data partition: A training set D was partitioned into K_2 non-overlapped parts where $(K_2 - 1)$ parts were used to train a model with hyper-parameter $\boldsymbol{\theta}_j$, which was then used to predict the held-out part and to compute the corresponding model performance metric (R^2 here); this process was repeated so that this step would give K_2 values of R^2.

Optimization: For a grid of J hyper-parameter combinations, denoted as $\boldsymbol{\theta}_j, j = 1, \ldots, J$, the same partition indices of K_2-fold CV were applied to each $\boldsymbol{\theta}_j$. Note $\boldsymbol{\theta}_j$ was either a vector (two parameters for eNet) or a scalar (other methods). The optimal hyper-parameter $\boldsymbol{\theta}_{\text{Opt}}$ were determined by the "one-standard-error" (1-SE) rule: among the $\boldsymbol{\theta}_j$'s whose mean R^2 (averaged across K_1 test sets) fell within one SE of the maximal mean R^2, the $\boldsymbol{\theta}_j$ that corresponded to the most parsimonious model was $\boldsymbol{\theta}_{\text{Opt}}$. Note that SE of the metric is defined as

$$SE = \frac{SD}{\sqrt{K_2}}$$

Should $\boldsymbol{\theta}_{\text{Opt}}$ be identified, the predicted values of the held-out sets at $\boldsymbol{\theta}_{\text{Opt}}$, denoted as $\hat{\boldsymbol{y}}(\boldsymbol{\theta}_{\text{Opt}})$, were extracted for stack ensemble.

Step ❷ Building a stack ensemble model. The predicted values of the optimal hyper-parameter $\boldsymbol{\theta}_{\text{Opt}}$ from L base learners (Step ❶) were then combined with the observed response values into a matrix $\widehat{\mathbf{D}}$ with $(L+1)$ columns. The observed response could be regressed on the m variables to obtain a stack ensemble model M^S. We chose to average predicted values of base learners weighted by their mean R^2 values from Step ❶.

Hyper-parameter selection. Elastic net had two tuning parameters α and λ. Both PCR and PLS had one parameter ("ncomp" for number of components). Both RF and CIF were tuned on a single parameter "mtry" for the number of predictors randomly selected to grow a tree; the number of trees was set at 500 and not tuned. Although in theory SVR may have three parameters: cost, ϵ, and σ, the scale parameter σ ("sigma") for radial basis function was estimated by the midpoint of the 10th and 90th percentiles of Euclidean distance between all training points (Caupto et al., 2002); also, the cost and ϵ parameters have some relationship and Kuhn suggests using cost ("C") as the only tuning parameter. To alleviate computation burden, we used random search (Bergstra and Bengio, 2012) with adaptive resampling algorithm (Kuhn, 2014) for no more than 15 parameter combinations, so the actually used parameter combinations and values varied with replications and training sets. Below, we present parameter values of a training set of a replicate as an example (the asterisk signs indicate the optimal parameters):

enet		PCR	PLS	rf	cforest	SVR	
alpha	lambda	ncomp	ncomp	mtry	mtry	sigma	C
0.101*	2.71E-05*	1	2*	1*	1	0.005*	0.154*
0.225	4.91E-04	2*	3	2	2*	0.006	9.581
0.241	1.57E-01	3	4	4	3	0.012	23.76
0.249	7.65E-05	5	6	5	4	0.013	6.299
0.297	2.78E-03	6	8	6	6	0.018	0.412
0.345	2.37E+00	8	11	7	7	0.022	546.3
0.433	3.21E-01	9	12	8	14	0.023	15.47
0.572	3.33E-05	11	13	9		0.031	39.52
0.824	3.57E-05	12		10		0.042	121.0
0.915	9.85E+00	13		11		0.103	72.47
0.917	1.26E-02					0.180	3.622
0.918	7.74E-02					0.181	21.61
0.919	5.72E-01					0.183	31.66
0.924	5.03E-04					0.339	0.788
0.970	8.59E-02					0.521	0.088

b. Mediation Analysis

We conducted path analysis (PA) modeling to explore potential mediation effects of respiratory measures (amplitude and rate) on the relations between motion and two most important predictors (Weight and Nicotine dependence). Respiration rate and amplitude were measured using a GE MR Bellows placed around the abdomen. Respiration rate was calculated by finding the number of peaks and dividing by total scan time. Respiration amplitude was calculated as the mean peak to trough difference, however, as this measure is related to the belt length rather than air flow, amplitude is in arbitrary units and is not equivalent to volume. Because weight and nicotine dependence were measured before scanning (pre-scan variables) while respiratory measures and motion were collected during scanning, it followed logic to use weight and nicotine dependence as independent variables, motion (log-transformed) as the dependent variable, and respiratory measures as potential mediators. These variables were first standardized to zero means and unit standard deviation before entering PA models. Due to 7 missing values in both respiratory amplitude and respiratory rate, we applied the full-information maximum likelihood estimation and robust (Huber-White) standard error for estimation. We began with a saturated model and removed non-significant paths one at a time. The final model was chosen to give a minimal value in sample-size adjusted-BIC statistic. This analysis was conducted using R lavaan package, version 0.5-23.1097 (Rosseel, 2012).

The final model contained 4 paths: from weight, nicotine dependence, and respiratory rate to motion, and from nicotine dependence to respiratory amplitude, and 1 covariance between respiratory amplitude and rate. The goodness-of-fit statistics included $\chi^2_{10} = 4.85\ (p = 0.90)$, CFI = 1.00, TLI = 1.04, RMSEA 0.00 (90% CI (0, 0.02)), SRMR 0.025. The path coefficients from weight to motion (0.293) and from nicotine dependence to motion (0.174) were only slightly different from path coefficients estimated in a model without respiratory variables (0.292 and 0.183, respectively). The small changes (0.3% and 4.9%, respectively) suggested negligible mediation effects of respiration.

2. Supplementary tables

Table S1: Characteristics of 464 subjects recruited for this study. BMI: Body Mass Index, PHQ-9: Patient Health Questionnaire, OASIS: Overall Anxiety Severity and Impairment Scale, DAST: Drug Abuse Screening Test.

	Healthy Control (57)	Mood/Anxiety (253)	Substance Use (154)	P Value
**Age** (mean(SD))	32.47 (11.16)	36.24 (11.08)	34.39 (9.26)	0.028
**Gender** (male (%))	28 (49.1)	74 (29.2)	74 (48.1)	<0.001
**Weight** (mean (SD))	178.35 (41.02)	179.54 (38.15)	185.04 (34.78)	0.298
**Height** (mean (SD))	67.28 (3.38)	66.33 (3.48)	67.47 (3.94)	0.006
**BMI**	27.62 (5.64)	28.63 (5.43)	28.49 (4.40)	0.403
**PHQ-9**	0.82 (1.20)	12.69 (5.11)	6.10 (5.44)	<0.001
**OASIS**	1.12 (1.39)	9.74 (3.46)	5.62 (4.59)	<0.001
**DAST**	0.12 (0.38)	0.64 (1.41)	7.61 (1.97)	<0.001

Table S2. 120 psychological (self-reports, non-drug related and drug related) and physical (age and body composition) variables with both univariate correlation (Pearson, r and P-value FDR corrected) with motion (log transformed) and variable importance (VI) in the stacked model. Right three columns represent the correlation and VI after regressing out the effect of physical variables (residualized (Res) r, p and VI)). None of the state and trait mood/anxiety/trauma related measures showed any significant correlation to the level of motion inside the scanner. Nicotine dependence is the only variable that survives FDR correction in the univariate correlation analysis with the motion after regressing out the effect of physical variables. Order of variables within each group (physical, drug related and non-drug related) are in alphabetic order.

Physical Variables	r	p	VI	r (Res)	p (Res)	VI (Res)
Age	0.173	0.002	43.31	0.087	0.404	NA
Body_Mass_Index_(BMI)	0.284	0	95.21	0.019	0.896	NA
Dry_Lean_Body_Mass	0.235	0	74.05	-0.006	0.96	NA
Fat_Body_Mass	0.195	0	59.21	-0.006	0.953	NA
Height	0.102	0.113	22.23	-0.02	0.883	NA
Hip_Size	0.168	0.003	37.37	-0.05	0.704	NA
Lean_Body_Mass	0.241	0	77.92	-0.03	0.841	NA
Percent_Body_Dry_Lean_	-0.07	0.334	26.05	0.022	0.862	NA
Percent_Body_Fat	0.061	0.453	24.99	-0.02	0.883	NA
Percent_Body_Water	-0.057	0.49	24.54	-0.032	0.841	NA
Sex	0.17	0.002	39.27	0.013	0.939	NA
Waist_Hip_Ratio	0.231	0	65.08	-0.039	0.841	NA
Waist_Size	0.283	0	84.41	0.047	0.728	NA
Water_Body_Mass	0.243	0	80.59	0.038	0.841	NA
Weight	0.303	0	98.5	0.007	0.952	NA

Self Reports, Drug Related Variables	r	p	VI	r (Res)	p (Res)	VI (Res)
Customary_Drinking_and_Drug_Use_Record_(CDDR)_Negative_Reinforcement_score	0.14	0.014	28.87	0.094	0.343	61.38
Customary_Drinking_and_Drug_Use_Record_(CDDR)_Positive_Reinforcement_score	0.078	0.261	13.91	0.067	0.583	22.24
Drug_Abuse_Screening_Test_(DAST-10)_score	0.134	0.019	29.8	0.112	0.163	44.2
PROMIS_Alcohol_Negative_Consequences_score	0.04	0.607	10.11	0.022	0.862	15.02
PROMIS_Alcohol_Negative_Expectancies_score	0.16	0.004	33.89	0.111	0.163	36.3
PROMIS_Alcohol_Positive_Consequences_score	-0.042	0.58	12.51	-0.032	0.841	32.73
PROMIS_Alcohol_Positive_Expectancies_score	0.02	0.811	12.65	0.028	0.841	23.13
PROMIS_Alcohol_Use_score	0.01	0.916	17.75	-0.008	0.951	28.84
PROMIS_Nicotine_Coping_score	0.158	0.005	33.81	0.13	0.148	74.26
PROMIS_Nicotine_Dependency_score	0.2	0	50.99	0.174	0.021	99.7
PROMIS_Nicotine_Emotional_and_Sensory_Expectancies_score	0.147	0.009	30.71	0.119	0.152	75.12
PROMIS_Nicotine_Health_Negative_Expectancies_score	0.193	0	41.36	0.135	0.148	71.26
PROMIS_Nicotine_Psychosocial_Expectancies_score	0.157	0.005	28.44	0.113	0.163	50.66
PROMIS_Social_Motivations_for_Nicotine_score	0.134	0.019	26.13	0.117	0.152	51.82

Self Reports, Non-Drug Related Variables	r	p	VI	r (Res)	p (Res)	VI (Res)
Anxiety_Sensitivity_Index_(ASI)_Cognitive_Concern_score	-0.042	0.58	10.62	-0.01	0.939	15.47
Anxiety_Sensitivity_Index_(ASI)_Physical_Concern_score	0.029	0.718	13.67	0.036	0.841	22.14
Anxiety_Sensitivity_Index_(ASI)_Social_Concern_score	-0.063	0.431	15.97	-0.052	0.703	22.9
Anxiety_Sensitivity_Index_(ASI)_Total_score	-0.033	0.672	12.73	-0.013	0.939	21.52
Behavioral_Approach_System_(BAS)_Drive_score	-0.004	0.964	8.72	-0.005	0.971	13.62
Behavioral_Approach_System_(BAS)_Drive_score	-0.042	0.58	9.92	-0.014	0.939	19.84
Behavioral_Approach_System_(BAS)_Fun_Seeking_score	0.075	0.287	16.24	0.091	0.385	32.93
Behavioral_Inhibition_System_(BIS)_Inhibition_score	-0.136	0.017	29.34	-0.096	0.327	30.33
Big_Five_Inventory_(BFI)_Agreeableness_score	0.031	0.689	12.34	0.07	0.556	30.19
Big_Five_Inventory_(BFI)_Conscientiousness_score	-0.061	0.453	14.48	-0.055	0.673	25.54
Big_Five_Inventory_(BFI)_Extraversion_score	0.043	0.58	14.16	0.055	0.673	26.84
Big_Five_Inventory_(BFI)_Neuroticism_score	-0.088	0.18	21.67	-0.088	0.404	47.9
Big_Five_Inventory_(BFI)_Openness_score	-0.051	0.56	14.27	-0.041	0.821	39.83
Childhood_Trauma_Questionnaire_(CTQ)_Denial_score	-0.055	0.508	11.01	-0.011	0.939	11.91
Childhood_Trauma_Questionnaire_(CTQ)_Emotional_Abuse_score	0.056	0.495	12.03	0.012	0.939	21.65
Childhood_Trauma_Questionnaire_(CTQ)_Emotional_Neglect_score	0.021	0.798	8.52	-0.028	0.841	20.29
Childhood_Trauma_Questionnaire_(CTQ)_Physical_Abuse_score	0.09	0.172	15.31	0.011	0.939	38.9
Childhood_Trauma_Questionnaire_(CTQ)_Physical_Neglect_score	0.049	0.567	9.14	-0.007	0.952	32.03
Childhood_Trauma_Questionnaire_(CTQ)_Sexual_Abuse_score	0.1	0.125	19.72	0.063	0.622	40.7
Childhood_Trauma_Questionnaire_(CTQ)_Total_score	0.08	0.244	13.63	0.015	0.939	33.74
Eating_Disorders_Diagnositic_Scale_(EDDS-3)_score	0.023	0.782	11.1	-0.028	0.841	30.42
International Physical Activity Questionnaire_(IPAQ)_Sitting_Minutes_score	-0.038	0.621	12.15	-0.054	0.673	41.6
International_Physical_Activity_Questionnaire_(IPAQ)_MET_Minutes_score	0.042	0.58	14.03	0.01	0.939	22.29
Interpersonal_Reactivity_Index_(IRI)_Empathic_Concern_score	-0.001	0.993	7.39	0.037	0.841	16.11
Interpersonal_Reactivity_Index_(IRI)_Fantasy_score	-0.093	0.161	15.91	-0.03	0.841	21.04
Interpersonal_Reactivity_Index_(IRI)_Personal_Distress_score	-0.082	0.236	15.34	-0.047	0.728	24.9
Interpersonal_Reactivity_Index_(IRI)_Perspective_Taking_score	-0.045	0.58	11	0.01	0.939	16.12
Multidimensional_Assessment_of_Interoceptive_Awareness_(MAIA)_Attention_Regulation_score	-0.048	0.577	10.3	-0.025	0.862	25.94
Multidimensional_Assessment_of_Interoceptive_Awareness_(MAIA)_Body_Listening_score	-0.05	0.56	11.5	-0.015	0.939	33.99
Multidimensional_Assessment_of_Interoceptive_Awareness_(MAIA)_Emotional_Awareness_score	-0.09	0.172	22.03	-0.05	0.704	53.8
Multidimensional_Assessment_of_Interoceptive_Awareness_(MAIA)_Not_Distracting_score	-0.046	0.58	11.95	-0.031	0.841	36.77
Multidimensional_Assessment_of_Interoceptive_Awareness_(MAIA)_Not_Worring_score	0.097	0.146	17.88	0.058	0.673	23.47
Multidimensional_Assessment_of_Interoceptive_Awareness_(MAIA)_Noticing_score	-0.036	0.646	8.34	0.001	0.979	19.33
Multidimensional_Assessment_of_Interoceptive_Awareness_(MAIA)_Self_Regulation_score	0.018	0.814	10.59	0.023	0.862	37.16
Multidimensional_Assessment_of_Interoceptive_Awareness_(MAIA)_Trusting_score	-0.023	0.782	9.07	-0.002	0.979	16.56
Overall_Anxiety_Severity_and_Impairment_Scale_(OASIS)_score	-0.034	0.67	10.97	-0.063	0.622	32.54
Patient_Health_Questionnaire_(PHQ-9)_score	-0.051	0.56	14.56	-0.078	0.464	32.24
Positive_and_Negative_Affect_Scale_(PANAS)_Attentiveness_score	0.009	0.919	12.2	-0.013	0.939	23.7
Positive_and_Negative_Affect_Scale_(PANAS)_Fatigue_score	-0.046	0.58	13.24	-0.031	0.841	18.04
Positive_and_Negative_Affect_Scale_(PANAS)_Fear_score	0	0.993	8.93	0.003	0.979	42.68
Positive_and_Negative_Affect_Scale_(PANAS)_Guilt_score	0.034	0.67	14.52	-0.01	0.939	26.68
Positive_and_Negative_Affect_Scale_(PANAS)_Hostility_score	-0.059	0.467	15.06	-0.081	0.447	32.75
Positive_and_Negative_Affect_Scale_(PANAS)_Joviality_score	0.043	0.58	13.33	0.06	0.639	21.35
Positive_and_Negative_Affect_Scale_(PANAS)_Negative_Affect_Total_score	-0.018	0.82	9.97	-0.026	0.854	34.74
Positive_and_Negative_Affect_Scale_(PANAS)_Positive_Affect_Total_score	0.043	0.58	13.79	0.043	0.807	23.71
Positive_and_Negative_Affect_Scale_(PANAS)_Sadness_score	-0.005	0.954	10.15	-0.038	0.841	28.92

Self Reports, Non-Drug Related Variables	r	p	VI	r (Res)	p (Res)	VI (Res)
Positive_and_Negative_Affect_Scale_(PANAS)_Self_Assurance_score	0.126	0.03	31.26	0.118	0.152	38.64
Positive_and_Negative_Affect_Scale_(PANAS)_Serenity_score	0.08	0.244	16.51	0.081	0.447	24.77
Positive_and_Negative_Affect_Scale_(PANAS)_Shyness_score	-0.039	0.62	11.14	-0.039	0.841	29.1
Positive_and_Negative_Affect_Scale_(PANAS)_Surprise_score	0.089	0.179	17.97	0.082	0.447	27.08
PROMIS_Ability_to_Participate_Social_Activities_score	0.009	0.916	12.94	0.049	0.713	24.77
PROMIS_Anger_score	-0.005	0.952	14.81	-0.029	0.841	32.27
PROMIS_Anxiety_score	-0.051	0.56	24.87	-0.047	0.728	39
PROMIS_Applied_Cognitive_Abilities_score	-0.018	0.814	15.3	-0.011	0.939	36.07
PROMIS_Applied_Cognitive_General_Concerns_score	-0.012	0.885	14.18	-0.031	0.841	21.54
PROMIS_Depression_score	-0.038	0.62	17.86	-0.074	0.502	26.04
PROMIS_Emotional_Support_score	0.015	0.855	13.29	0.061	0.622	35.32
PROMIS_Fatigue_score	-0.008	0.929	16.18	-0.022	0.862	16.56
PROMIS_Informational_Support_score	0.013	0.872	12.43	0.063	0.622	29.52
PROMIS_Interest_in_Sexual_Activities_score	0.141	0.013	31.54	0.118	0.152	81.79
PROMIS_Pain_Behavior_score	0.086	0.2	21.52	0.056	0.673	33.35
PROMIS_Pain_Interference_score	0.07	0.334	20.68	0.036	0.841	36.58
PROMIS_Physical_Function_score	-0.104	0.11	24.4	-0.029	0.841	19.8
PROMIS_Positive_Affect_and_Wellbeing_score	0.047	0.577	15.81	0.08	0.451	31.23
PROMIS_Sleep_Disturbance_score	0.006	0.942	17.1	-0.034	0.841	16.95
PROMIS_Sleep_Related_Impairment_score	-0.007	0.929	12.88	-0.004	0.975	17.26
PROMIS_Social_Isolation_score	-0.027	0.755	13.31	-0.067	0.583	22.63
PROMIS_Social_Satisfaction_in_Participation_in_Descretionary_Social_Activities_score	-0.023	0.782	17.17	0.013	0.939	27.7
PROMIS_Social_Satisfaction_with_Role_score	0.018	0.814	14.58	0.075	0.5	36.6
Ruminative_Response_Scale_(RRS)_score	-0.032	0.683	12.89	-0.056	0.673	38.06
SickControlOneFatFood_Questionnaire_(SCOFF)_score	0.014	0.868	7.69	-0.03	0.841	22.54
State_Trait_Anxiety_Inventory_(STAI)_State_score	-0.049	0.567	13.41	-0.062	0.622	33.29
State_Trait_Anxiety_Inventory_(STAI)_Trait_score	-0.035	0.665	12.99	-0.05	0.704	24.34
Temporal_Experience_of_Pleasure_Scale_(TEPS)_Anticipatory_score	-0.024	0.782	7.91	0.028	0.841	17.83
Temporal_Experience_of_Pleasure_Scale_(TEPS)_Consummatory_score	0.044	0.58	12.07	0.071	0.538	20.58
Three_Factor_Eating_Questionnaire_(TEFQ)_Diet_Restraint_Score	-0.16	0.004	33.64	-0.126	0.152	43.79
Three_Factor_Eating_Questionnaire_(TEFQ)_Disinhibition_Score	-0.019	0.814	9.65	-0.099	0.304	30.5
Three_Factor_Eating_Questionnaire_(TEFQ)_Hunger_Score	0.043	0.58	9.35	-0.023	0.862	12.72
Torento_Alexithymia_Scale_(TAS)_Difficulty_in_Describing_Feelings_score	-0.002	0.978	8.73	-0.027	0.849	29.48
Torento_Alexithymia_Scale_(TAS)_Difficulty_in_Identifying_Feelings_score	0.014	0.868	9.93	-0.002	0.979	18.39
Torento_Alexithymia_Scale_(TAS)_Externally_Oriented_Thinking_score	0.058	0.485	15.67	0.054	0.673	38.58
Torento_Alexithymia_Scale_(TAS)_Total_score	0.028	0.746	10.77	0.008	0.951	31.68
Traumatic_Events_Scale_(TES)_Total_Occurance_score	0.091	0.172	18.11	0.001	0.979	17.51
Traumatic_Events_Scale_(TES)_Total_score	0.106	0.098	27.32	0.028	0.841	33.49
Traumatic_Events_Scale_(TES)_Total_Worst_Intensity_score	0.07	0.334	14.9	0.042	0.807	19.92
UPPS_Impulsive_Behavior_Scale_(UPPS-P)_Lack_of_Perseveration_score	0.022	0.791	10.12	0.028	0.841	21.48
UPPS_Impulsive_Behavior_Scale_(UPPS-P)_Lack_of_Premediation_score	0.152	0.007	29.15	0.131	0.148	57.68
UPPS_Impulsive_Behavior_Scale_(UPPS-P)_Negative_Urgency_score	0.071	0.333	14.24	0.023	0.862	15.75
UPPS_Impulsive_Behavior_Scale_(UPPS-P)_Positive_Urgency_score	0.141	0.013	29.07	0.086	0.404	27.02
UPPS_Impulsive_Behavior_Scale_(UPPS-P)_Sensation_Seeking_score	0.095	0.156	18.98	0.077	0.464	34.47
WHO_Disability_Assessment_Screen_(WHODAS)_total_score	0.025	0.782	11.93	-0.023	0.862	34

Table S3. Pearson correlation coefficients between variables used in mediation analysis

	Motion	Weight	Nicotine dependence	Respiratory Amplitude	Respiratory Rate
Motion		0.30<br>P < 0.0001	0.20<br>P < 0.0001	0.16<br>P = 0.0007	-0.23<br>P < 0.0001
Weight			0.06<br>P = 0.21	0.05<br>P = 0.31	0.00<br>P = 1.00
Nicotine dependence				0.11<br>P = 0.015	-0.04<br>P = 0.39
Respiratory Amplitude					-0.33<br>P < 0.0001

Table S4. Path coefficients of mediation analyses

Model	Path	Estimate	Std. Err.	Z statistic	p-value
No reparatory	Wt to Mot	0.292	0.046	6.404	< 0.0001
	NcD to Mot	0.183	0.046	4.008	< 0.0001
Final model	Wt to Mot	0.293	0.044	6.629	< 0.0001
	NcD to Mot	0.174	0.043	4.000	< 0.0001
	Rate to Mot	-0.219	0.041	-5.324	< 0.0001
	NcD to Amp	0.100	0.046	2.167	0.030

Wt: Weight; Mot: motion; NcD: nicotine dependence; Rate: respiratory rate; Amp: respiratory amplitude

3. Supplementary figures

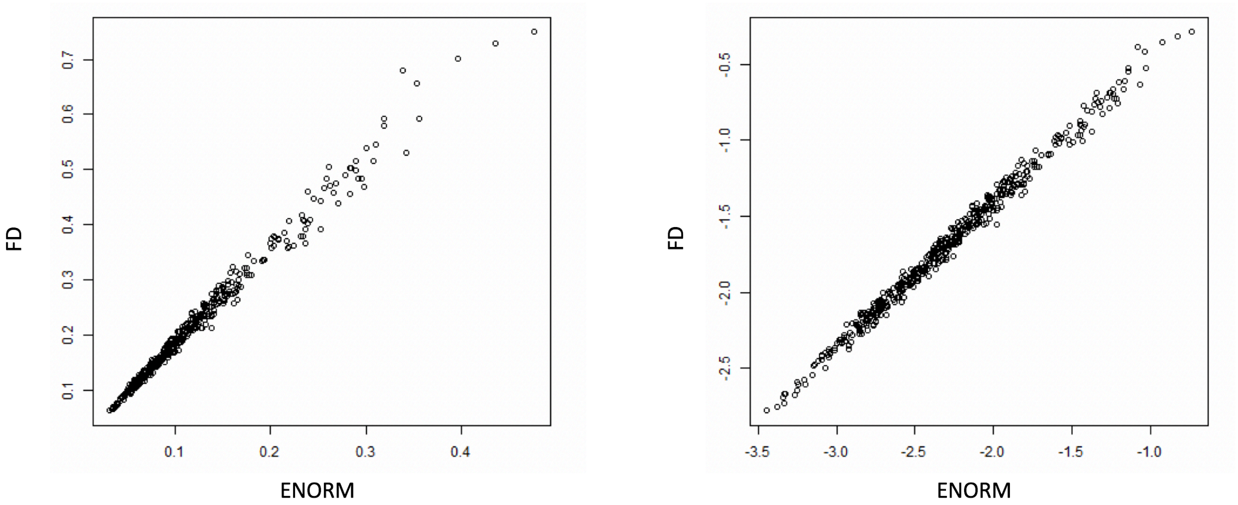

Figure S1. Scatterplots between two measures of motion, Euclidean Norm (ENORM, average Euclidean norm of six motion parameters) and Framewise Displacement (FD, sum of the absolute values of the six motion parameters) without and with natural log transformation (r=0.993 and 0.995 consecutively) (n=464).

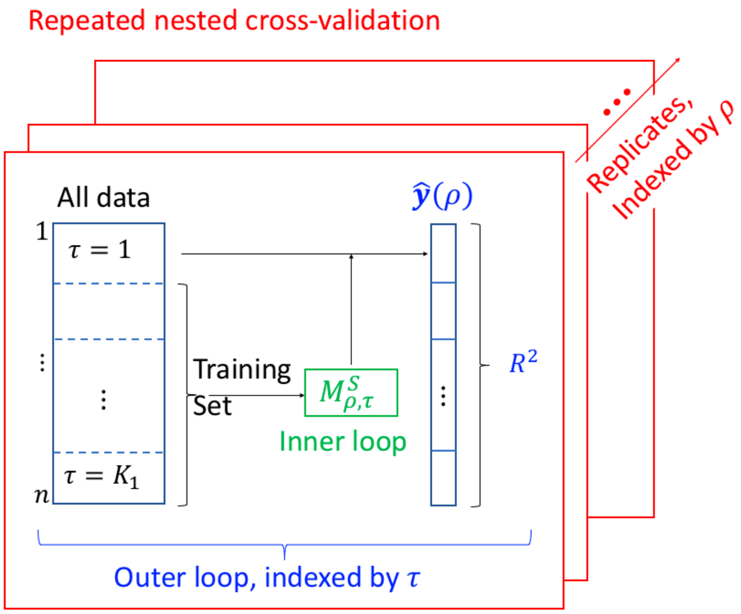

Figure S2(a). The process of repeated nested CV. This figure illustrates the process of nested CV repeated 20 times indexed by ρ. In the outer loop, the original data of n subjects were partitioned into K_1 parts indexed by τ. For each iterate, $(K_1 - 1)$ parts served as a training set to optimize tuning parameters in the inner loop (see Supplementary Figure 1b.). The stack model $\mathrm{M}^S_{\rho,\tau}$ from the inner loop was then used to predict the held-out set of the outer loop. Predicted values $\hat{\boldsymbol{y}}(\rho)$ were combined across held-out sets to evaluate the performance of the stack models.

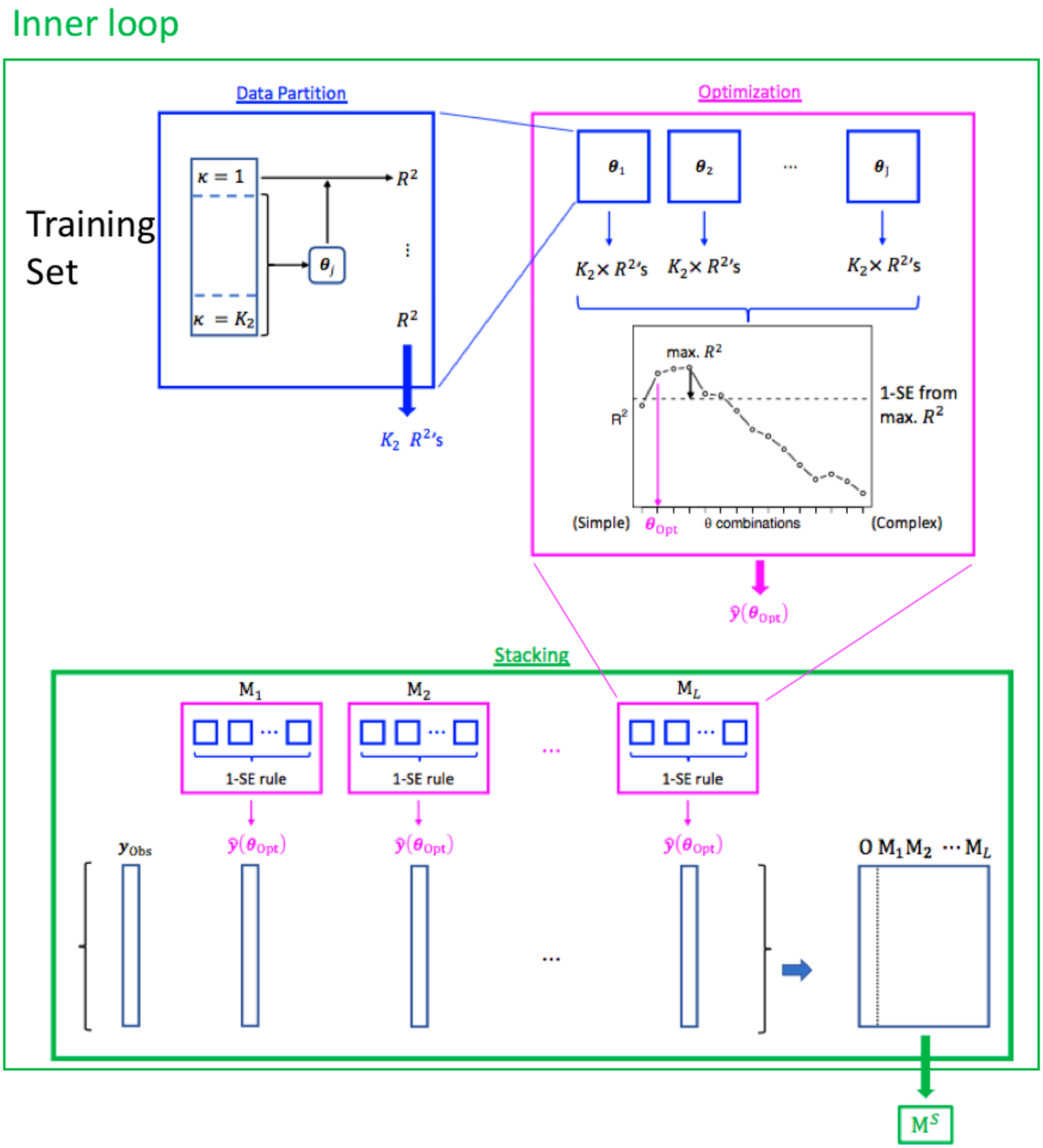

Figure S2(b). The inner-loop of repeated nested CV. In the inner loop, each training set from the outer loop was further partitioned into K_2 parts indexed by κ. A model with a tuning parameter combination $\boldsymbol{\theta}_j$ was trained on $(K_2 - 1)$ parts, and then this model was applied to predict the held-out set. The predicted values were then compared to the observed values by R-square. Iterating across K_2 folds led to K_2 values of R-square. The process was repeated for all parameter combinations, and the optimal tuning parameter combination ($\boldsymbol{\theta}_{\mathrm{Opt}}$) was determined by the "one-SE" rule. This optimal parameter combination was applied to the whole training set and gave the predicted values $\hat{\boldsymbol{y}}(\boldsymbol{\theta}_{\mathrm{Opt}})$. This process was then repeated for all machine learning methods. The predicted values across machine learning methods were then combined with observed values to build a stack model M^S.

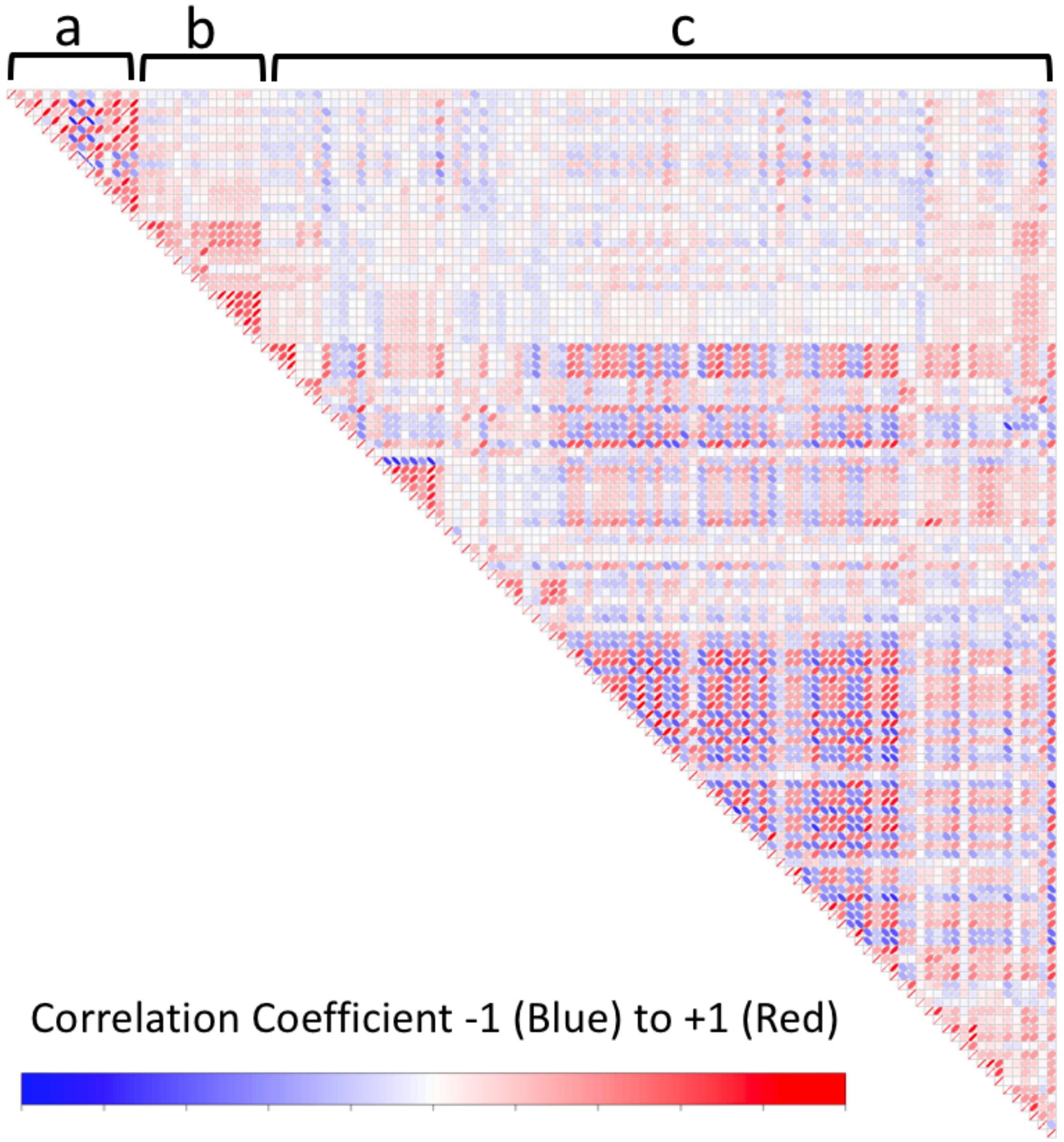

Figure S3. Correlation matrix between 120 variables to explain motion representing high levels of correlation among variables. a: Physical variables, b: Drug-related psychological variables, and, c: Non-drug-related psychological variables

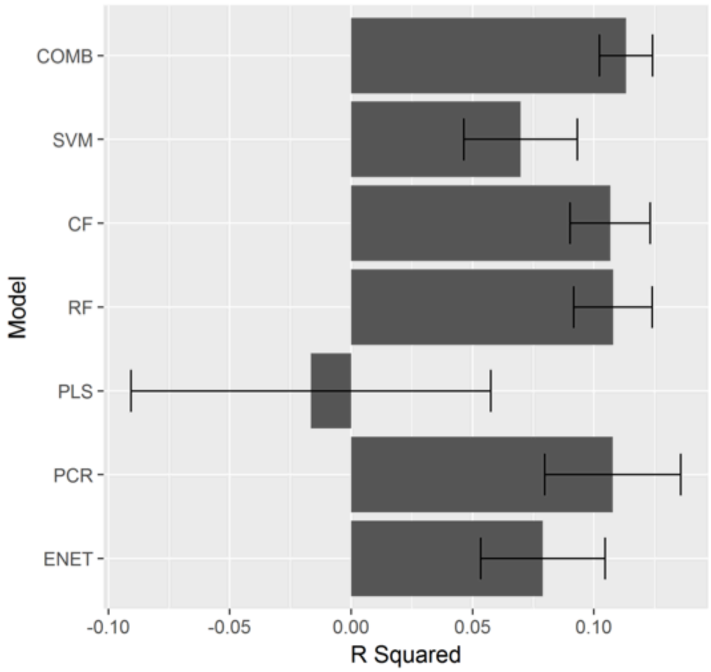

Figure S4. Percent of variance explained for motion with all variables using different methods. ENET: Elastic net, PCR: Principle component regression, PLS: Partial least square, RF: Random forest, CF: Conditional forest, SVM: Support vector machine, COMB: Combined (stacked) method. Error bars represent 95% confidence intervals.

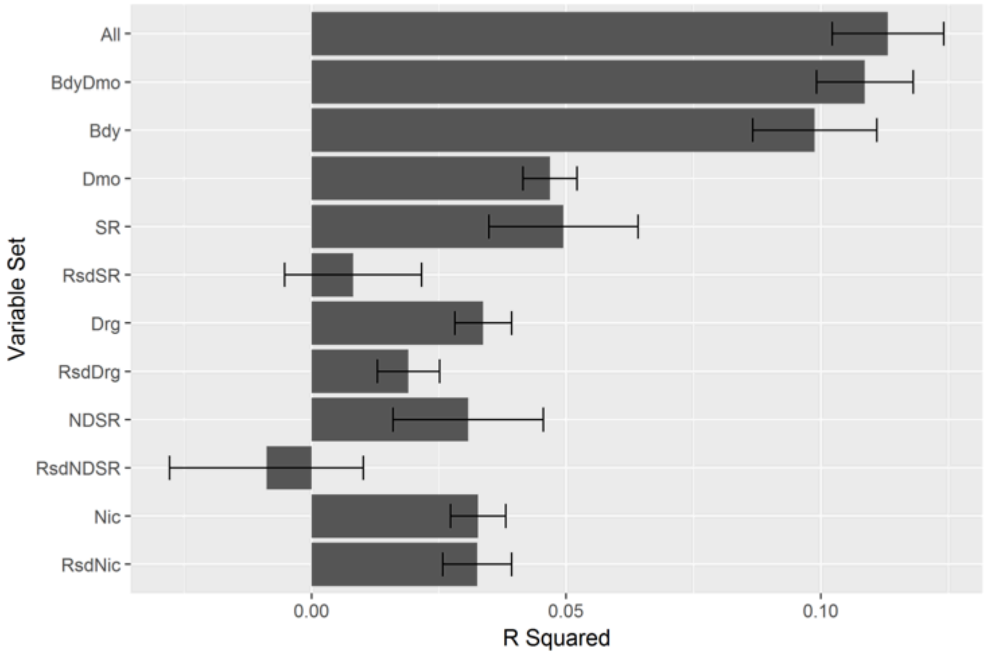

Figure S5. Percent of variance explained for motion with different sets of variables using combined (stacked) method. Bdy: Anthropometrics, Dmo: Demographics, SR: Self Reports, Rsd: Residualized after regressing out the Bdy and Dmo variables, Drg: Drug (including alcohol and

nicotine) related self reports, NDSR: Non-drug related self report, and Nic: Nicotine-related self reports. Error bars represent 95% confidence intervals.

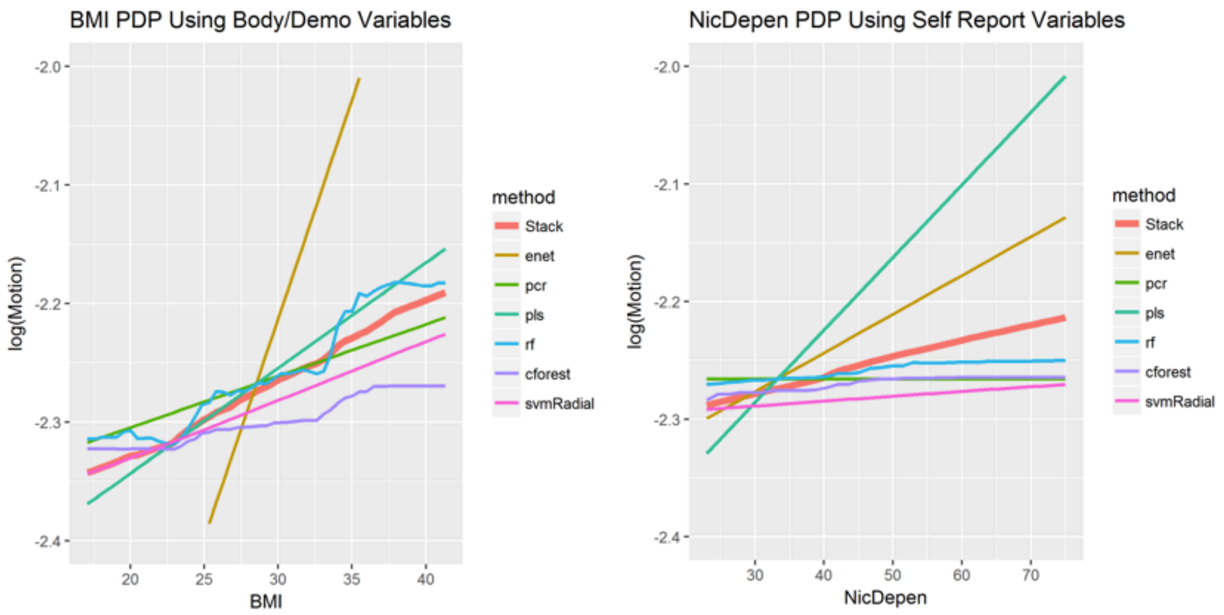

Figure S6. Partial dependence plots for the variables with the highest importance among anthropometric (a, BMI) and self-report (b, NicDepen) variables. Individual models vary, but agree in direction where coefficients are non-zero. The COMB model appears as a weighted average of the others, and is somewhat between the most extreme individual models. ENET: Elastic net, PCR: Principle component regression, PLS: Partial least square, RF: Random forest, CF: Conditional forest, SVM: Support vector machine, COMB: Combined (stacked) method.

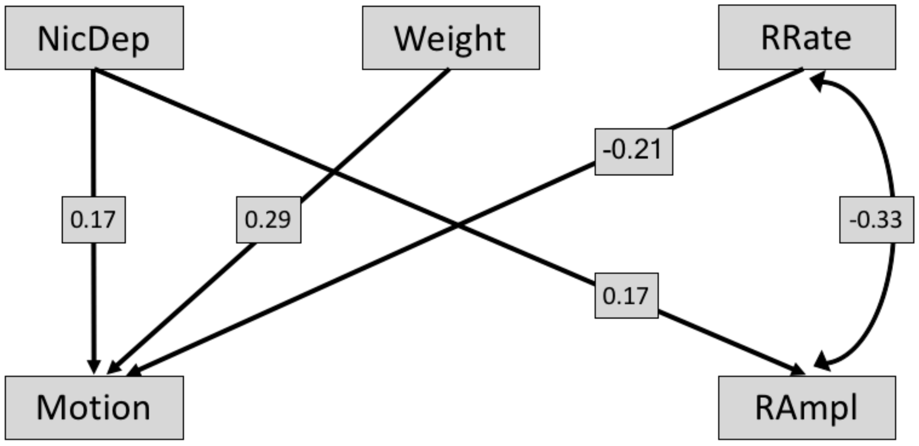

Figure S7. Path diagram of the final mediation model. The values indicate path coefficients (straight arrows) and covariance (arc) for standardized variables. RRate: respiratory rate, RAmp: respiratory amplitude, and NicDep: PROMIS Nicotine Dependency score.

Reference

Bergstra J and Bengio Y. Random search for hyper-parameter optimization. Journal of Machine Learning Research. 2012: 13: 281 – 305.

Caputo B, Sim K, Furesjo F, Smola A (2002). “Appearance–Based Object Recognition Using SVMs: Which Kernel Should I Use?” In “Proceedings of NIPS Workshop on Statistical Methods for Computational Experiments in Visual Processing and Computer Vision,”

Filzmoser P, Liebmann B, Varmuza K. Repeated double cross validation. Chemometrics. 2009. 23: 160 – 171.

James G, Witten D, Hastie T, & Tibshirani R. (2013). *An introduction to statistical learning: With applications in R.* Springer-Verlag New York.

Kuhn M. (2014). Futility analysis in the cross–validation of machine learning models. (unpublished).

Kuhn, M., & Johnson, K. (2013). *Applied predictive modeling*. New York, NY: Springer.

Rosseel Y. (2012). lavaan: An R Package for Structural Equation Modeling. *Journal of Statistical Software*, 48(2), 1-36.

Stone M: Cross-validatory choice and assessment of statistical predictions. J Roy Stat Soc B Met 1974, 36, No. 2:111–147.

Varma S and Simon R. Bias in error estimation when using cross-validation for model selection. BMC Bioinformatics, 2006, 7:9.
